# Lactobacilli-Host mutualism: "learning on the fly"

**DOI:** 10.1186/1475-2859-13-S1-S6

**Published:** 2014-08-29

**Authors:** Renata C Matos, François Leulier

**Affiliations:** 1 Institut de Génomique Fonctionnelle de Lyon (IGFL), Ecole Normale Supérieure de Lyon, CNRS UMR 5242, Université Claude Bernard Lyon 1, 46 Allée d'Italie, 69364 Lyon Cedex 07, France

**Keywords:** Microbiota, *Lactobacillus*, *Drosophila*, Mutualism

## Abstract

Metazoans establish with microorganisms complex interactions for their mutual benefits. *Drosophila*, which has already proven useful host model to study several aspects of innate immunity and host-bacteria pathogenic associations has become a powerful model to dissect the mechanisms behind mutualistic host-microbe interactions. *Drosophila *microbiota is composed of simple and aerotolerant bacterial communities mostly composed of *Lactobacillaceae *and *Acetobactereaceae. Drosophila *mono- or poly-associated with lactobacilli strains constitutes a powerful model to dissect the complex interplay between lactobacilli and host biologic traits. Thanks to the genetic tractability of both *Drosophila *and lactobacilli this association model offers a great opportunity to reveal the underlying molecular mechanisms. Here, we review our current knowledge about how the *Drosophila *model is helping our understanding of how lactobacilli shapes host biology.

## Introduction

Metazoans establish with microorganisms complex interactions for their mutual benefits. When the host-bacteria system is in balance or homeostasis, it can contribute for many aspects of host physiology [1]. As an example, the gut bacterial communities or microbiota improve the digestion of nutrients and provide new metabolic functions to their host. They also contribute to the education and stimulation of the immune system and are responsible for colonization resistance against pathogens [2]. On the bacterial side, host is furnishing a niche with accessibility to substrates either exogenous from food or endogenous from mucus. However, when the tight balance between host and microbiota is broken it can result in the development of certain pathologies [1].

**Figure 1:**
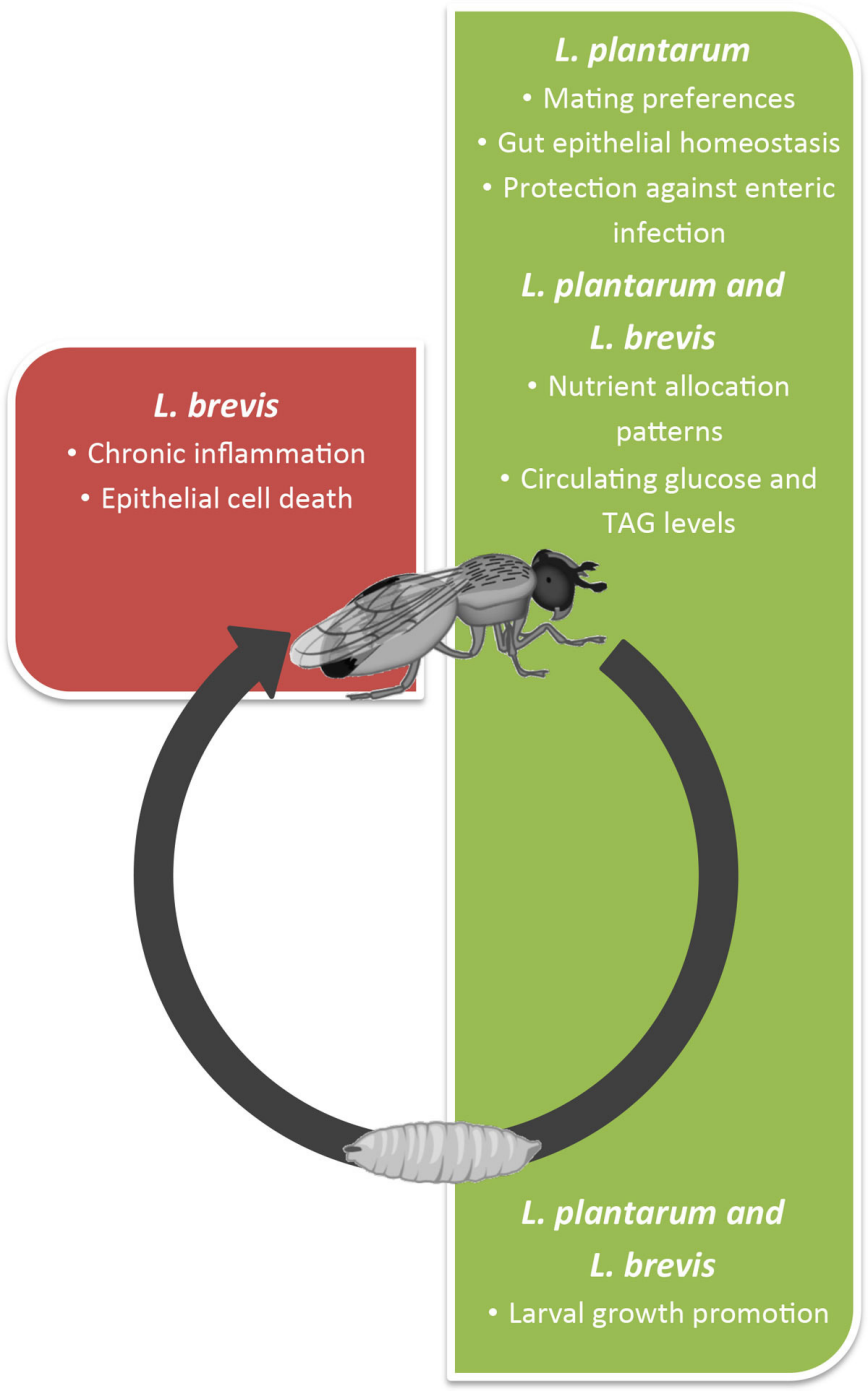
**Functional impact of commensal lactobacilli on *Drosophila *biology**. Strains of *L. plantarum *can be true mutualists and impact different aspects of host biology: mating preferences, gut epithelial homeostasis, protection against enteric infection, nutrient allocation and metabolism. During larval stages it promotes systemic growth. Depending on the strain and host genetic background, *L. brevis *is a mutualist of pathobiont, which interferes with host metabolism and/or induces chronic inflammation and epithelial cell death.

Over the last 15 years, the composition of the gut microbiota has been explored by culture-independent techniques [3] and further described by metagenomic analysis [4,5]. Analyses of the gene encoding 16S rRNA by pyrosequencing have identified up to ten phyla represented in microbiota. At a lower taxonomic level, up to a thousand species are generally present in one individual. Metagenomic analysis has revealed, within the inter-individual variation, the presence of less than sixty species shared among human individuals [5]. The relative proportion of each species is influenced by environmental and host factors [6]. Despite the ongoing interest on the gut microbiota, the mechanisms behind the interactions between host and microbes remain to be better understood. This comprehension is hampered by the complexity and variability of the bacterial communities involved in mammalian host-microbe interactions. In addition, most of the bacteria of the human gastrointestinal tract have not yet been cultured *ex-vivo*, due in part to their anaerobic metabolism or sensitivity to oxygen; those that have been cultured require laborious techniques [7]. Thus, the use of simpler animal models may help to unravel evolutionary conserved mechanisms underlying the impact of intestinal bacterial in their host physiology. In this light, *Drosophila*, which has already proven useful in the study of several aspects of innate immunity and host-bacteria pathogenic associations [8], has become a powerful model to dissect the mechanisms behind mutualistic host-microbe interactions (Figure 1) [9,10]. *Drosophila *combines genetic and experimental tractability with a culturable microbiota of low diversity that facilitates microbial genetic analysis.

## *Drosophila *gut microbiota composition: high prevalence of lactobacilli

Several studies have determined the composition of *Drosophila *gut commensal bacterial communities and the factors that shape their diversity and relative abundance. Overall *Drosophila *microbiota is composed of simple bacterial communities represented by the phyla *Firmicutes *(*Lactobacillaceae *and *Enterococcaceae*) and *Proteobacteria *(*Acetobactereaceae *and *Enterobactereaceae*) with five dominating species: *Acetobacter pomorum, A. tropicalis, Lactobacillus brevis, L. plantarum *and *L. fructivorans *[11,12]. With the exception of *Acetobactereaceae*, these species are also commensals in humans [5]. Strains of *Gluconobacter morbifer *and more recently *L. brevis *were identified as *Drosophila *pathogens that under certain circumstances can act as a colitogenic pathobionts due to their ability to constantly release uracil [11,13]. Uracil release by pathogenic bacteria and certain opportunistic pathobionts was identified as the ligand inducing DUOX-dependent reactive oxygen species generation in *Drosophila*'s gut, which is required for the efficient elimination of bacteria but also causes inflammation and cell death. In the case of these opportunistic pathobionts, the permanent release of uracil and subsequent DUOX activation leads to chronic inflammation [13].

Studies to determine *Drosophila *gut microbiota composition were carried out in whole flies or dissected guts from laboratory-raised and wild-caught flies. The diversity was determined using both culturable-dependent and culture-independent techniques such as amplification and sequencing of microbial 16S rRNA. Globally they report that *Drosophila *gut bacterial communities are sensitive to variations on diet, developmental stage and host immune status [11,12,14,15].

Clearly, the most important factor shaping *Drosophila*'s gut microbiota is the diet. Corby-Harris investigated community richness and composition of 11 fly populations recovered from different latitudes. Although the authors observed significant spatial variation in microbial community richness they could not find a clear relationship between latitude or climate and microbial richness [16]. Likewise, the analysis of natural bacterial communities associated with different species of *Drosophila *collected from distant geographical locations revealed that the gut microbiota composition of different species feeding on the same type of substrates were more similar to each other then to more closely related species feeding on different substrates [14,17]. In those studies the differences in microbial content were attributed to food composition. Supporting these observations, it was shown that increasing the carbohydrate to protein ratio in the medium enhances the proportion of *Acetobacter *versus *Lactobacillus *in young adult flies [12,18] and that changes on diet such as high-fat or short- term starvation have drastic and long-lasting effects on the microbiota [19]. Although the major represented groups detected in laboratory-raised flies were found to be present in most of wild *Drosophila *populations, lab-reared flies and wild-caught flies diverge in their microbiota composition. Moreover, bacterial communities associated with lab-reared flies are striking less diverse than those of wild-caught flies. Thus, so far, it was not possible to define a group of microorganisms consistently recovered from *Drosophila *that we could call a core microbiota [14,15,17]. Nevertheless it is reasonable to think that taxonomically variable bacterial communities can be functionally equivalent and instead of a core microbiota, *Drosophila *would sustain a core microbiome.

Other factors shaping *Drosophila's *gut microbiota composition are developmental status and aging. Indeed, a case study from the Douglas' lab indicated that despite bacterial species presence all along *Drosophila's *life cycle, their abundance varies with developmental age. During larval growth the dominant species in the fly line they studied changes from *L. fructivorans *to *L. plantarum*. In the same line, at pupal stages *A. tropicalis *was the most represented species while in young adults, *L. fructivorans *became dominant again and in old adults *A. pomorum *was the dominant species in the community [12]. It has been suggested that the gut oxidative status could be driving species predominance during *Drosophila*'s life cycle favouring either aerobic or aerotolerant bacteria growth or it may reflect different nutritional needs and immune performances [9,12]. In fact, old flies have their bacterial loads increased, which has been associated to age-related decrease in the efficiency of immune responses [20,21]. However the global composition and abundance of the gut microbiota in *Drosophila *is extremely variable among different laboratories and among *Drosophila *adults within the same line [17], so this issue yet deserves further investigation. Finally, host genetic determinants can also impact the density and composition of gut bacterial communities, as has been demonstrated by Ryu and colleagues [11]: flies with higher antimicrobial peptides production due to a mutation on the homeobox gene *Caudal *(*Cad*) show a shift in community composition from the commensal bacteria *Commensalibacter intestini *to a minor member of *Drosophila's *gut, *Gluconobacter morbifer*, recognized as a pathobiont.

Despite the numerous studies on *Drosophila*'s gut microbiota composition and the general agreement of the community in that the most represented species are member of the genus *Lactobacillus* and *Acetobacter*, their global composition and abundance remains extremely variable among wild-caught and lab-raised flies as well as between and within different laboratories over time [17]. Moreover, a recent study by Blum and colleagues determine how *Drosophila*'s gut microbiota is established and maintained. They evaluate bacterial communities of newly emerged flies transferred to fresh food daily for 3 days or kept in the same food for 7 days and realise that flies that were not transferred harboured larger bacterial populations than those that were transferred. In this way they confirmed that *Drosophila *need to consume bacteria from the environment in order to establish and maintain its microbiota, which indicate that bacteria do not persist long in *Drosophila*'s gut [22]. These two aspects should be taken into account when working with *Drosophila *since they can have an impact on observed phenotypes.

As mentioned before, lactobacilli are prevalent commensals of *Drosophila melanogaster *similarly to mammals including humans. In *Drosophila*, they reside in the intestine and are vertically transmitted to progenies via the deposition of contaminated mother's faeces on the surface of the embryo during egg laying and on the surrounding substratum. Many strains of different lactobacilli species (*L. plantarum, L. brevis, L. fructivorans*) can colonize germ-free animals and remained associated to their host during its entire life cycle by constant re- association through ingestion. In addition to being commensal species of *Drosophila melanogaster *intestine, lactobacilli have been used for decades as a model lactic acid bacteria and therefore offers vast technical resources and potential [23]. Therefore the approach of using lactobacilli and an animal host model such as *Drosophila *with evolutionary conserved genetic and physiological features to study intestinal host-microbe interactions is well suited to unravel lactobacilli/host interactions encountered in the wild potentially including those occurring in humans.

## Functional impact of lactobacilli on *Drosophila *biology

### Impact on host behaviour

Sharon and colleagues investigated if diet could impact fly's behaviour such as mating preferences. Flies reared in cornmeal-molasse-yeast medium (CMY, a high carbohydrate to protein ratio diet containing simple sugars) were split in two groups: one group was kept in CMY medium whereas the other was shifted to starch medium (lower carbohydrate/protein ratio without addition of simple sugars). After few generations they were transferred to mating chambers and tested for mating preferences [24]. Flies are more sexually attracted to individuals fed on the same diet showing assortative mating (non-random). This preference seems to be dependent on the gut microbiota composition. Indeed, starch diet-fed flies harbour 10 times more lactobacilli than CMY-fed flies (confirming the strong impact of diets on microbiota composition). This sexual preference was abolished by antibiotic treatment since flies behaviour passed from positive assortative to random after treatment. The analysis of the bacterial communities associated with flies reared in each medium revealed a strong association of flies reared on starch diet with *L. plantarum*. Subsequent experiments revealed a preference of ex-germ-free, *L. plantarum *monoassociated flies to mate with starch-diet fed flies (carrying high prevalence of *L. plantarum*) in detriment of CMY-fed flies [24]. The analysis of the cuticular hydrocarbon (communication clues for insects) composition of axenic flies and conventional flies reared in CMY and starch medium showed significant differences among flies harbouring different gut microbiota. All together the results from this work suggest that symbiotic bacteria, and in particular *L. plantarum *can influence mating preferences by changing the levels of cuticular hydrocarbon sex pheromones establishing a link between environment (diet), microbiome and behaviour. However, the exact mechanism by which bacteria influence mating preferences remains to be elucidated [24]. Similarly, a recent paper by the Dukas' lab showed that *Drosophila *larvae associated to lactobacilli (*L. brevis *and *L .plantarum *strains) significantly attracted adult flies or other larvae to their hospitable nutritional niche but were non attractive to others if they lacked a microbiota [24,25]. These observations suggest again that the host microbiota mediates behavioural responses, yet the underlying mechanism remains elusive but the *Drosophila *model which has historically been the premier model system for understanding the molecular and genetic bases of complex behaviours [26] offers great promise to identify them.

### Protection against infection

A major function of the gut microbiota is the protection against colonization by pathogens and the control of pathobionts overgrowth. It was already observed that certain members of the fly gut microbiota provide protection against pathobiont overgrowth [11]. Recently, Blum and colleagues proposed that the gut microbiota can also protect the fly against infection by a pathogen [22]. Conventional (CONV) and germ-free flies associated with a strain of *L. plantarum *were less susceptible than germ-free animals to gut infection by *Serratia marcescens*. The authors suggest that the protection is correlated with *L. plantarum *density in the flies that is higher in associated ex-germ-free flies than associated-CONV flies. In order to enlarge the number of species tested, the authors evaluated the level of protection given by *Enterococcus faecalis *and conclude that it does not protect flies against infection by these pathogenic bacteria. Although only one strain of *L. plantarum *and *E. faecalis *were tested, the authors claim that the protective effect seems *L. plantarum *specific. Further studies with higher number of strains should be conducted to better elucidate the specificity of the phenotype as well as determine how *L. plantarum *exerts its protective effect [22]. Given the interest of using lactobacilli as human probiotics, the authors evaluated the protective effect of *Lactobacillus rhamnosus *(which is not a *Drosophila *commensal) against *S. marcescens *and *Pseudomonas aeruginosa*. As *S. marcescens *is a fly and human pathogen and strains of *L. plantarum *and *L. rhamnosus *are used as probiotics in humans and had a protective effect against infections in *Drosophila*, the authors suggest that the *Drosophila*-lactobacilli association model might be useful to unravel probiotic effects of different lactobacilli strains since they may be conserved and further translated to humans [22]. This seems a valuable model, however more complete and detailed studies need to be performed in support of these exciting preliminary observations.

### Gut epithelial homeostasis

In order to maintain homeostasis, the gut epithelium is continuously replenished by stem cells. The disruption of this equilibrium may result in disease [27]. In *Drosophila*, midgut cells are replenished by a population of intestinal stem cells (ISCs) which division generates a new ISC and a post-mitotic enteroblast that differentiates into an adsorptive enterocyte or a secretory enteroendocrine cell [28]. Buchon and colleagues established for the first time that the gut microbiota stimulates a basal level of stem cell activation and subsequent epithelium renewal since axenic animals present slower intestinal epithelial cell renewal rate than conventional flies [21]. The similarities between *Drosophila *and mammals ISCs can promote the genetic analysis of normal and abnormal intestinal function in a simpler model and can help to elucidate the role of the microbiota in gut homeostasis [28].

The work by Jones and colleagues further characterize how the gut microbiota affects epithelial renewal and identify that lactobacilli is particularly prone to do so [29]. The authors first demonstrated that the association of germ-free *Drosophila *with *L. plantarum *induces ROS generation by midgut enterocytes. Association with other members of the gut microbiota did not elicit the same response. Since *L. plantarum*, a commensal bacteria of the *Drosophila's *gut, is able to induce ROS production in the intestine, the authors investigate the involvement of dNOX and dDUOX for this phenotype, the only two NADPH oxidases in *Drosophila*. Through the midgut specific expression of dsRNA targetting dNOX and dDUOX the authors identified that *L. plantarum *induced ROS production is dependent on dNOX.

Finally they determined that *L. plantarum *induce ROS-dependent cellular proliferation in the *Drosophila *intestine [29]. It is interesting to note that a previous work by Lee and colleagues detected DUOX-dependent ROS production induced by pathogenic bacteria and pathobionts in *Drosophila*'s gut [13]. It is hard to integrate both results since the time of detection and the compounds used to detect ROS production were not the same. In fact, Lee and colleagues detected a stronger form of ROS, HOCl, and Jones and colleagues used a compound that detects a broader range of ROS [13,29]. Further studies should conciliate these two sets of data.

In sum, the authors demonstrated that lactobacilli are strong inducers of endogenous ROS generation and ROS-dependent cellular proliferation within *Drosophila *intestines but also mammals. These two effects are dependent on a functional Nox enzyme in intestinal epithelial cells. It was demonstrated with this work that lactobacilli-induced ROS generation is conserved in metazoans, which strengths the use of *Drosophila *simple model to study intestinal homeostasis since the mechanisms are probably conserved in mammals [29]. Future research should target the molecular mechanisms behind lactobacilli promotion of epithelial homeostasis in both bacteria and host.

### Impact on nutrition and related metabolism

The nutritional status of animals is deeply influenced by the gut microbiota that can provide supplementary nutrients to the host, alter nutrient assimilation and allocation patterns [30]. Several recent studies addressed the question of how *Drosophila *gut microbiota shapes host nutritional traits [18,31,32].

Ridley and colleagues compared host performance, nutritional status and metabolic rate of conventional flies harboring a microbiota dominated by *A. pomorum *and axenic flies. This work revealed that axenic flies have higher glucose, trehalose and glycogen contents than conventional flies demonstrating that the gut microbiota impacts the carbohydrate allocation patterns of adult *Drosophila *[18]. Very recently Wong and colleagues investigated the combine effect of diet and absence of the intestinal microbiota on *Drosophila *performance and nutrient allocation [32]. The authors showed that the intestinal microbiota supports *Drosophila *performance on diets of low or unbalanced nutrient content but also that it is implicated in vitamin B and protein nutrition as well as energy storage [32].

To understand the contribution of individual microbial strains and interactions between strains in shaping nutrient allocation, Newell and Douglas tested strains of the 5 most common species in *Drosophila*'s intestinal microbiota (*A. pomorum, A. tropicalis, L. brevis, L. plantarum *and *L. fructivorans*) in monoassociation or poly-association with *Drosophila *[31]. The authors compared conventional flies, 5-species microbiota associated flies and flies monoassociated with each one of the 5 strains in several nutritional parameters: adult weight and protein, glucose, glycogen and TAG contents. All 5 species reduced circulating glucose levels to that of conventional flies. Concerning TAG content, none of the conditions with lactobacilli recapitulated conventional flies content, nevertheless *L. plantarum *treatment reduced slightly TAG content when compared with axenic flies. Flies monoassociated with *Acetobacter *species showed reduced TAG levels but only the 5-species microbiota recapitulate conventional flies TAG content, which suggests interaction among species for this host phenotype. Those results led the authors to test the impact of all possible pairwise *Acetobacter*-*Lactobacillus *combinations on nutritional parameters. Interestingly, the pair *A. tropicalis*-*L. brevis *treatment reduced TAG to levels significantly lower than 5-species microbiota revealing a very strong effect of this bacterial association. Likewise, this pair was also the most efficient in lowering flies weight. The authors suggest that *Acetobacter*- mediated reduction of host TAG is promoted by co-colonization with *Lactobacillus *species, which revels a phenotype that is dependent on interspecies interaction inside *Drosophila*'s intestine that needs to be further studied [31].

The studies mentioned above confirmed the impact of *Drosophila *gut microbiota on several aspects of host nutrition, including nutrient allocation patterns and highlights the importance of gut microbiota composition in host metabolic phenotypes.

### Impact on host development

Several studies have addressed the impact of *Drosophila *intestinal microbiota for systemic growth and unravel some of the mechanism underlying this phenotype [18,31,33,34]. Globally, those studies revealed that on a conventional diet rich in simple sugars and proteins, axenic flies emerge approximately 2 days later than conventional flies. However, under nutrient scarcity such developmental delay is massively increased [18,31,33,34]. Clearly, *Drosophila *systemic growth is influenced by nutrient availability as well as intestinal microbiota composition. Thus the presence of gut microbiota should be particularly useful under harsh condition that flies might face in the wild. In order to narrow down the effects of the microbiota on *Drosophila *systemic growth to a specific member of the consortium or interactions amongst individual species, Newell and Douglas evaluated the contribution of strains of the 5 most common species in *Drosophila*'s intestinal microbiota (*A. pomorum, A. tropicalis, L. brevis, L. plantarum *and *L. fructivorans*) to host developmental time [31]. They evaluated the effect of the defined 5-strains microbiota (poly-association) and the effect of each strain individually upon mono-association with *Drosophila*. Both mono-association of *A. pomorum *and *A. tropicalis *are able to produce developmental times comparable to those of the 5-strains gut microbiota whereas *L. plantarum *and *L. brevis *have an intermediate effect and *L. fructivorans *has no visible effect [31]. It should be noted that as the diet influences gut microbiota composition and activity, which fluctuates depending on carbohydrates to protein ratio and levels of simple sugars, the higher level of simple sugars in the diet used in Newell's paper might have hampered the prevalence and activity of lactobacilli and therefore their impact on *Drosophila*'s growth. Indeed, *Acetobacter *predominates on simple sugar rich diet compare to lactobacilli [33,34]. Two other studies revealed the contribution of *Drosophila *microbiota to its host systemic growth [33,34]. Shin and colleagues, using a simple sugar containing diet, revealed the marked impact of strains of *Acetobactereaceae *species (*Acetobacter pomorum, Commensalibacter intestini *and *Gluconobacter morbifer*). They also identified the growth promoting effect, albeit less marked than *Acetobactereaceae*, of *Lactobacillus plantarum *and *L. brevis *strains. Then, Shin *et al*. mainly worked on a monoassociation *Acetobacter*-*Drosophila *model and evidenced both host and bacterial factors underlying growth promotion effect but also energy metabolism and intestinal stem cell activity [33]. In fact, production of acid acetic by the bacterial pyrroloquinonoline quinone- dependent alcohol dehydrogenase (PQQ-ADH) modulates insulin/insulin-like growth factor signalling (IIS) in *Drosophila *leading to the control of host nutritional signalling networks. However more bacterial functions might be involved since the simple addition of acetic acid to the medium did not restore the growth promotion effect [33]. In the case of Storelli and colleagues, standard fly diets containing inactivated yeast and starch from cornmeal and devoid of simple sugar addition were used and *L. plantarum*, the most prevalent bacterial species in these flies (no *Acetobactereaceae *were detected) and was able to promote larval growth upon nutrient scarcity as efficiently as a control microbiota resulting in early adult emergence [34]. *L. plantarum *beneficial effect is strain specific since other strains were not able to promote larval growth in similar conditions pointing to the existence of specific bacterial activities present in growth promoting strains that non-growth promoting strains lack which are essential for the *Drosophila *growth phenotype to express [34]. *L. plantarum *exerts its beneficial effect on larval growth through the host nutrient sensing system that relies on the tissue specific activity of the TOR kinase that subsequently modulates hormonal signals controlling growth and maturation. Stimulation of TOR kinase activity by diet-derived branched-chain amino acids in the fat body leads to increased *Drosophila *insulin-like peptides (dILPs) production by the brain [35]. At the same time, activation TOR kinase activity in the prothoracic gland, promotes Ecdysone production during late larval stage and impacts on the length of the growth phase [34]. Efforts in our lab are on-going to decipher how *L. plantarum *promotes TOR activity; *L. plantarum*-mediated enhanced digestion and absorption of peptides from the diet leading to optimization of diet-derived branched-chain amino acids in the hemolymph is the current working hypothesis.

## Conclusions

*Drosophila melanogaster *has emerged as a powerful model to study host-gut microbiota mutualism given the simplicity and low-complexity of its microbial communities and the ease to generate and maintain gnotobiotic animals. *Drosophila *mono- or poly-associated with lactobacilli strains under certain nutritional conditions constitutes a powerful model to dissect the complex interplay between diet, bacteria and host biologic traits and thanks to the genetic tractability of both *Drosophila *and lactobacilli this model offers a great opportunity to reveal the underlying molecular mechanisms. Given its contribution to unravel TOR and IIS pathways involvement in *Lactobacillus plantarum *mediated growth promotion, this model will also help dissecting if and how other lactobacilli strains, including available probiotic strains promote juvenile growth and/or influence host metabolism paving the way to the use, the identification or the design of next generation evidence-based probiotic strains. In addition, given its pioneer role in the study of the fundamental roots of innate immunity and host/pathogen interactions [8], the *Drosophila *model may also offer opportunities to study the interplay between lactobacilli, metabolism and the innate immune system. Finally, studies on the *Drosophila *microbiota/gut/brain axis [36] and its impact on host behaviour [24,25] offer exciting and refreshing perspectives to the lactobacilli/Host interaction field.

## List of abbreviations

rRNA: ribosomal ribonucleic acid; CMY: cornmeal-molasse-yeast; ISC: intestinal stem cell; ROS: reactive oxygen species; TAG: triglyceride; IIS: insulin/insulin-like growth factor signalling; PQQ-ADH: pyrroloquinonoline quinone-dependent alcohol dehydrogenase; TOR: target of rapamycin; dILPs: insulin-like peptides.

## Competing interests

The authors declare that they have no competing interests.
